# Mechanistic science in cardiovascular-oncology: the way forward to maximise anti-cancer drug effects and minimise cardiovascular toxicity

**DOI:** 10.1042/CS20210986

**Published:** 2021-12-09

**Authors:** Ninian N. Lang, Rhian M. Touyz

**Affiliations:** 1 Institute of Cardiovascular and Medical Sciences, University of Glasgow, Glasgow, Scotland; 2 Research Institute of the McGill University Health Centre, McGill University, Montreal, Canada

**Keywords:** cancer, cardiovascular disease, pharmacology

## Abstract

Dramatic improvements in cancer survival have arisen because of the rapid development of novel anti-cancer therapies. The potential for cardiovascular toxicity associated with these drugs often reflects overlap between pathogenic cancer mechanisms and physiological pathways required for normal cardiovascular function. *Clinical Science* has, therefore, compiled a themed collection on *Cardiovascular-Oncology*. This collection examines the intersection between cancer treatments and their potentially harmful cardiovascular effects. By defining the mechanisms underlying unwanted cardiovascular effects of anti-cancer therapies, cardioprotective strategies can be developed. Only by doing so, will patients be able to achieve optimal cancer treatment at the minimum cost to cardiovascular health.

Recent decades have seen remarkable improvements in cancer survival. In the context of this success in cancer treatment, minimising the potential for competing cardiovascular risks has never been more important. Indeed, *Cardiovascular-Oncology* is now well-established as a clinical specialty and is receiving an appropriate increase in attention from the pre-clinical scientific community. Patients with cancer are more prone to cardiovascular comorbidity as a result of shared genetic predispositions [1,2] and risk factors including smoking, obesity and inflammation [2]. Furthermore, this heightened risk for cardiovascular disease may be further amplified by cancer therapies because of overlap between pathways required for normal cardiovascular homoeostasis and those involved in tumour initiation, growth and metastasis. Many targeted anti-cancer therapies have the potential to cause cardiovascular adverse effects by acting simultaneously upon physiologic and pathogenic pathways and mediators. Furthermore, there has been an associated reinvigoration to improving our understanding of the mechanisms and strategies to mitigate cardiovascular toxic effects of conventional chemotherapeutic agents, principally anthracyclines. In this themed collection on *Cardiovascular-Oncology*, these areas are addressed with particular focus on mechanistic pathways relevant to cardiovascular toxicity of anti-cancer therapies.

## Targeted therapies

Although the association between anthracycline exposure and subsequent left ventricular dysfunction and heart failure has been recognised since the 1960s [3], the introduction of trastuzumab for the treatment of HER2-positive breast cancer prompted much more widespread concern from cardiologists. In early trials of patients treated with this monoclonal antibody directed against ErbB/neuregulin-1 (NRG-1), excellent anti-cancer effects were observed, the likes of which had not been seen before in this aggressive tumour subgroup, but with a substantial incidence of cardiotoxicity (27% when given concomitantly with an anthracycline [4]; 7.2% in meta-analysis of subsequent trials [5]). By virtue of these clinical observations, reverse translation identified the important role of NRG-1 in myocardial physiology [6]. More recently, targeting tumour angiogenesis has become an important strategy in the treatment of many solid organ cancers, including renal, hepatocellular, thyroid and others. Vascular endothelial growth factor (VEGF) signalling is the primary target of these anti-angiogenic drugs. However, in contrast with the early situation with trastuzumab, the importance of VEGF in cardiovascular haemostasis and development had been well described for decades prior to therapeutic targeting. It is, therefore, not surprising that anti-VEGF drugs are strongly associated with unwanted cardiovascular effects including hypertension [7,8] and left ventricular dysfunction [9] which, at least partly, reflects microvascular dysfunction. It is notable that other therapies, including targeted inhibition of rapidly accelerated fibrosarcoma B-type (BRAF) and mitogen-activated extracellular signal-regulated kinase (MEK) for the treatment of cutaneous melanoma are also associated with a broad range of toxicities [10]. This is not unexpected given the well-known and important role of these mediators of the mitogen activated protein kinase (MAPK) signalling cascade in cardiovascular physiology. There is now a much greater appreciation that adverse effects of anti-cancer therapies extend far beyond ‘just’ left ventricular dysfunction and that a complex interplay exists between micro- and macro-vascular diseases with hypertension, direct myocardial toxicity and frequently a pro-inflammatory, pro-atherogenic environment [9].

## Immunotherapy

Immunotherapy has provided impressive advances in cancer survival, including for patients with advanced and aggressive malignancies who would previously have had the most dismal of prognoses. In those patients who respond to immunotherapy, radiographic disappearance of advanced cancer may be achieved and survival for many years is possible. In a recent analysis, almost 40% of patients with cancer would currently be eligible for treatment with immunotherapy [11]. While the majority of treatment-related adverse effects are non-cardiovascular, myocarditis can be a devastating complication, with mortality at approximately 50% in early case series and registries. A range of other cardiovascular adverse effects is becoming clearer and includes atherogenesis and myocardial infarction as well as non-inflammatory cardiomyopathy, vasculitis and pericarditis [12]. Furthermore, there is growing evidence for the use of immunotherapy in combination with angiogenesis (VEGF) inhibitors. The cardiovascular consequences of more prolonged exposure to angiogenesis inhibition remain unknown, although these are a concern and require careful prospective ascertainment via longer term trial follow-up with appropriately defined cardiovascular endpoints and via dedicated cardio-oncology registries. Whether angiogenesis inhibition potentiates the cardiovascular toxic effects of immunotherapy, particularly atherogenesis and plaque inflammation remains unclear [13]. In more general terms, there is great potential for interaction between cardiovascular therapies and anti-cancer agents, especially via cytochrome p450 effects [14].

## Anthracyclines

The growth of cardiovascular-oncology has stimulated better understanding of the cardiotoxic effects and mechanisms associated with anthracyclines. Attempts to prevent anthracycline-induced left ventricular dysfunction using conventional neurohormonal therapies including β-blockers and renin–angiotensin system inhibitors have been somewhat disappointing [15]. The established benefits of these drugs are in patients with the clinical syndrome of heart failure and consequent neurohormonal system activation. It is highly conceivable that a different approach targeting the direct myocardial toxic effects of anthracycline exposure would be more effective for the prevention or treatment of asymptomatic left ventricular dysfunction [16]. Dexrazoxane, an iron chelating agent with additional effects upon topoisomerase 2β, is approved for use in a narrow range of circumstances for the prevention of anthracycline-induced cardiotoxicity. However, better mechanistic understanding and alternative approaches would be valuable. The effects of a novel pro-drug (ICRF-193) has been elegantly explored by Kollárová-Brázdová and colleagues in a rabbit model of anthracycline-induced cardiotoxicity [17]. Carrera and colleagues provide an overview of the potential for manipulation of cytochrome 1B1 for the prevention of cancer therapy-associated cardiac dysfunction while simultaneously having the potential to sensitise anti-cancer effects, including in the context of anthracyclines [18].

## The future

Clinical and basic science endeavours in cardiovascular-oncology have, historically, been reactive to the large and rapid successes in cancer treatment but are finally becoming increasingly pro-active. As a community, cardiologists and cardiovascular researchers must work in closer collaboration with oncologists and cancer scientists and pay particular attention to defining the mechanisms underlying unwanted cardiovascular effects of anti-cancer therapies. Only by doing so, will patients be able to achieve optimal cancer treatment at the minimum cost to cardiovascular health.

## Abbreviations

NRG-1: neuregulin-1
VEGF: vascular endothelial growth factor

## References

[1] Sionakidis A., McCallum L. and Padmanabhan S. (2021) Unravelling the tangled web of hypertension and cancer. Clin. Sci. 135, 1609–1625 10.1042/CS20200307 34240734

[2] Mooney L., Goodyear C.S., Chandra T. et al. (2021) Clonal haematopoiesis of indeterminate potential: intersections between inflammation, vascular disease and heart failure. Clin. Sci. 135, 991–1007 10.1042/CS20200306 PMC8055963 33861346

[3] Tan C., Tasaka H., Yu K., Murphy M.L. and Karnofsky D.A. (1967) Daunomycin, an antitumor antibiotic, in the treatment of neoplastic disease. Clinical evaluation with special reference to childhood leukemia. Cancer 20, 333–353 10.1002/1097-0142(1967)20:3<333::AID-CNCR2820200302>3.0.CO;2-K 4290058

[4] Slamon D.J., Leyland-Jones B., Shak S. et al. (2001) Use of chemotherapy plus a monoclonal antibody against HER2 for metastatic breast cancer that overexpresses HER2. N. Engl. J. Med. 344, 783–792 10.1056/NEJM200103153441101 11248153

[5] Chen T., Xu T., Li Y. et al. (2011) Risk of cardiac dysfunction with trastuzumab in breast cancer patients: A meta-analysis. Cancer Treat. Rev. 37, 312–320 10.1016/j.ctrv.2010.09.001 20952131

[6] Lemmens K., Doggen K. and Keulenaer G.W.D. (2007) Role of Neuregulin-1/ErbB signaling in cardiovascular physiology and disease. Circulation 116, 954–960 10.1161/CIRCULATIONAHA.107.690487 17709650

[7] Neves K.B., Montezano A.C., Lang N.N. and Touyz R.M. (2020) Vascular toxicity associated with anti-angiogenic drugs. Clin. Sci. 134, 2503–2520 10.1042/CS20200308 32990313

[8] van Dorst D.C.H., Dobbin S.J.H., Neves K.B. et al. (2021) Hypertension and prohypertensive antineoplastic therapies in cancer patients. Circ. Res. 128, 1040–1061 10.1161/CIRCRESAHA.121.318051 33793337 PMC8011349

[9] Dobbin S.J.H., Petrie M.C., Myles R.C., Touyz R.M. and Lang N.N. (2021) Cardiotoxic effects of angiogenesis inhibitors. Clin. Sci. 135, 71–100 10.1042/CS20200305 PMC7812690 33404052

[10] Welsh S.J. and Corrie P.G. (2015) Management of BRAF and MEK inhibitor toxicities in patients with metastatic melanoma. Ther. Adv. Med. Oncol. 7, 122–136 10.1177/1758834014566428 25755684 PMC4346212

[11] Haslam A., Gill J. and Prasad V. (2020) Estimation of the percentage of US patients with cancer who are eligible for immune checkpoint inhibitor drugs. JAMA Netw. Open 3, e200423 10.1001/jamanetworkopen.2020.0423 32150268 PMC7063495

[12] Baik A.H., Tsai K.K., Oh D.Y. and Aras M.A. (2021) Mechanisms and clinical manifestations of cardiovascular toxicities associated with immune checkpoint inhibitors. Clin. Sci. 135, 703–724 10.1042/CS20200331 PMC8647663 33686402

[13] van Dorst D.C.H., van Doorn L., Colafella K.M.M. et al. (2021) Cardiovascular toxicity of angiogenesis inhibitors and immune checkpoint inhibitors: synergistic anti-tumour effects at the cost of increased cardiovascular risk? Clin. Sci. 135, 1649–1668 10.1042/CS20200300 34283204

[14] Kamaraju S., Mohan M., Zaharova S. et al. (2021) Interactions between cardiology and oncology drugs in precision cardio-oncology. Clin. Sci. 135, 1333–1351 10.1042/CS20200309 PMC8984624 34076246

[15] Vaduganathan M., Hirji S.A., Qamar A. et al. (2019) Efficacy of neurohormonal therapies in preventing cardiotoxicity in patients with cancer undergoing chemotherapy. JACC Cardio Oncol. 1, 54–65 10.1016/j.jaccao.2019.08.006 33083790 PMC7571368

[16] Narezkina A., Narayan H.K. and Zemljic-Harpf A.E. (2021) Molecular mechanisms of anthracycline cardiovascular toxicity. Clin. Sci. 135, 1311–1332 10.1042/CS20200301 PMC10866014 34047339

[17] Kollárová-Brázdová P., Lenčová-Popelová O., Karabanovich G. et al. (2021) Prodrug of ICRF-193 provides promising protective effects against chronic anthracycline cardiotoxicity in a rabbit model in vivo. Clin. Sci. 135, 1897–1914 10.1042/CS20210311 34318878

[18] Carrera A.N., Grant M.K.O. and Zordoky B.N. (2020) CYP1B1 as a therapeutic target in cardio-oncology. Clin. Sci. 134, 2897–2927 10.1042/CS20200310 PMC7672255 33185690

